# Response Surface Methodology for Ni-Zeolite Catalyst Optimization in Syngas Production

**DOI:** 10.1021/acsomega.4c05617

**Published:** 2024-09-25

**Authors:** Yousef M. Alanazi, Ahmed S. Al-Fatesh, Fahad S. Al-Mubaddel, Ahmed A. Ibrahim, Anis H. Fakeeha, Ahmed E. Abasaeed, Najib Y. A. AL-Garadi, Ahmed I. Osman

**Affiliations:** † Chemical Engineering Department, College of Engineering, King Saud University, Riyadh 11421, Saudi Arabia; ‡ School of Chemistry and Chemical Engineering, Queen’s University Belfast, Belfast, BT9 5AG Northern Ireland, U.K.

## Abstract

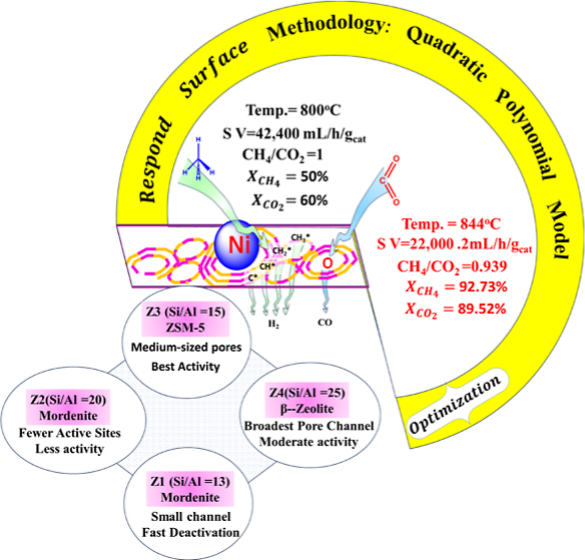

This work addresses the problem of converting waste methane, a significant greenhouse gas, using customized nickel-zeolite catalysts to produce profitable syngas. The investigation employs 5 wt % of Ni on various zeolite supports with Si/Al ratios ranging from 13 to 25. Comprehensive characterization methods, including temperature-programmed reduction, N_2_ adsorption–desorption, and X-ray diffraction, were used to identify critical structural characteristics that greatly impact the catalyst’s performance. The study indicates that the reducibility and basicity of the catalyst, the type of zeolite support, and the kind of carbon deposits formed during the reaction at 800 °C all influence the efficiency of methane conversion to syngas. The best catalyst was found to be 5Ni-Z3, which at 800 °C produced high conversion rates of carbon dioxide (60%) and methane (50%). Lastly, the response surface methodology, in conjunction with numerical simulation, was used to determine the best operating settings for maximizing syngas production with the 5Ni-Z3 catalyst. Reaction temperature, space velocity, and the methane-to-carbon dioxide feed ratio were considered in this analysis. With a methane conversion rate exceeding 92%, a carbon dioxide conversion rate exceeding 90%, and a hydrogen-to-carbon monoxide ratio of 1.00, the catalyst produced experimental results very similar to the SRM predictions when the reaction was conducted at conditions close to the predicted values [temperature around 845 °C, space velocity around 22,000 mL/(h·gcat), and feed ratio close to 0.94]. The effectiveness of the identified operating conditions for the dry reforming process is validated by the near alignment of expected and experimental outcomes.

## 1 Introduction

Nanomaterials have transformed the field of catalysis, offering unique properties and enhanced performance due to their nanoscale dimensions. Among these, zeolites have gained much attention because of their well-defined pore structures, high surface areas, and tunable acidity.^1^ These unique characteristics make them ideal supports for catalytic applications, enabling improved dispersion of active metal sites and enhanced catalytic activity, which are used in multiple environmental applications, among which is climate change mitigation. The deadly impact of global warming is now seen everywhere in the form of heat waves, drastic seasonal changes, record rises in sea levels, etc.,^2^ and these conditions worsen year after year.^3^ The major cause of this scenario are the greenhouse gases CH_4_ and CO_2_. In this run, the catalytic conversion of dry reforming of methane (DRM) has drawn major attention, which converts CH_4_ and CO_2_ into syngas (H_2_ and CO). The industrial implications of DRM are depletion of the concentration of both greenhouse gases as well as formation of valuable synthetic feedstock syngas. After syngas, hydrogen serves as a versatile energy route, finding applications as a transportation fuel and a raw material for various chemical industry sectors, including the production of synthetic fuels such as ammonia, methanol, and synthetic liquid fuels.^4^ The conventional industrial process for hydrogen production is the steam reforming of methane.

However, an alternative approach, such as DRM, presents itself through the direct use of biogas as the feedstock, utilizing both methane and carbon dioxide.^5−9^Eq 1 displays the DRM reaction. DRM is limited by its endothermic nature, which requires elevated reaction temperatures, causing various side reactions to occur, particularly methane decomposition, CO disproportionation, and reverse water–gas shift (RWGS) reaction.^6^ Three primary side reactions accompanying the DRM reaction are listed in eqs 2–4
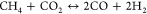
1
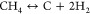
2
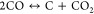
3
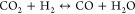
4

Because of their desirable properties of being more readily available and less expensive than catalysts based on noble metals, nickel-based catalysts have gained significant attention. However, the stability of Ni-based catalysts is often suppressed by metal sintering and carbon deposition, leading to major catalyst deactivation.^9−13^ DRM reactions typically run within the temperature range of 700–1000 °C to thermodynamically restrict the RWGS side reactions and coking, too.^14,15^ Nonetheless, these elevated temperatures develop issues related to sintering and catalyst deactivation. To address this limitation, materials resistant to coke formation are employed as supports.^7,16^ Well-dispersed Ni catalysts with a consistent microporous surface and a characteristic tiny pore size can be made with porous support materials like zeolites.^17^ Xie et al. have created Ni catalysts for methane reforming that are successfully encased in silica. They discovered that the optimum catalyst with good Ni particle distribution was produced using a particular preparation technique with a medium Ni concentration.^18^ A study to reform CH_4_ was performed to produce syngas.^19^ The optimal Ni concentration was 5%, underscoring the significance of the metal–catalyst interaction in DMR catalyst optimization.^20^ An innovative DRM catalyst addresses a persistent issue: deactivation. Zeolite and Ni–Co alloy nanoparticles are combined in this one-pot system. The zeolite stabilizes the catalyst and improves the CO_2_ conversion, while the alloy increases the C–H bond breaking. This breakthrough opens the door to a more sustainable and effective use of CH_4_.^21^ Therefore, zeolites could provide the porous support material for Ni to aid metal dispersion and enhance the catalytic activity for the production of syngas.^22−25^ A thorough knowledge of how distinct zeolite features affect Ni catalyst behavior in DRM is still elusive, even though several techniques, including changing the support material, have been investigated to improve catalyst performance and stability.^16^ The stability and performance of the Ni catalyst can be affected by several important zeolite support-related parameters.^26,27^ The zeolite’s pore size and dimensionality may impact coke formation and metal sintering. Coking can be lessened by adjusting the distribution of acid sites and the Si/Al ratio.^28^ Catalyst longevity depends on how simple it is to remove coke deposits from the zeolite by oxidative renewal. Preferred zeolites have strong thermal stability and little propensity to dealumination during regeneration.^29^ Supported Ni catalysts with several zeolites are tested to evaluate the financially viable options for producing hydrogen.^30,31^ Besides adjusting the catalyst components, manipulating process parameters such as gas hourly space velocity, reaction temperature, and feed composition is a practical way to achieve the best catalytic DRM performance. Optimization through response surface methodology involves using multivariate designs to assess the interactions between input and output variables to determine the best conditions.

In this article, we will conduct a comparative study to investigate the impact of 5 wt % Ni supported on different types of zeolites (Z1, Z2, Z3, and Z4) with Si/Al ratios of 13, 20, 15, and 25, respectively. The study will focus on the catalytic activity and stability in the CO_2_ reforming of methane under varying reaction temperatures and an activation temperature of 800 °C. We will also optimize the input process parameters (reaction temperature, space velocity, and feed gas ratio—CH_4_/CO_2_) for the DRM process using a 5 wt % Ni supported on a Z3 zeolite catalyst. The response surface methodology was used for optimization. Once the optimized reaction conditions are identified, the predicted values from the simulation model are compared to the actual experimental values. Physicochemical characterization methods, including N_2_-physisorption, TPR, CO_2_-TPD, X-ray diffraction (XRD), TEM, Raman spectroscopy, and temperature gravimetric analysis (TGA), will be utilized to understand the properties of the materials.

## 2 Materials and Methods

### 2.1 Catalyst Preparation

5Ni/Zx catalysts are made, where Zx refers to types of zeolites: Z1, Z2, Z3, and Z4. The supports (Z1, Z2, Z3, and Z4) have BET surface areas of 324, 361, 305, and 471 m^2^/g, with molar ratios of SiO_2_ to Al_2_O_3_ of 13, 20, 15, and 25, respectively. 10 mL of distilled water and 5% Ni were combined and stirred at room temperature in an 80 mL glass crucible. The Zx support was then added, and the solution was dried with stirring for 30 min at 80 °C. The catalysts were calcined in air at a temperature of 600 °C using a heating rate of 3 °C/h for up to 3 h.

### 2.2 Catalytic Analysis

**Figure 1:**
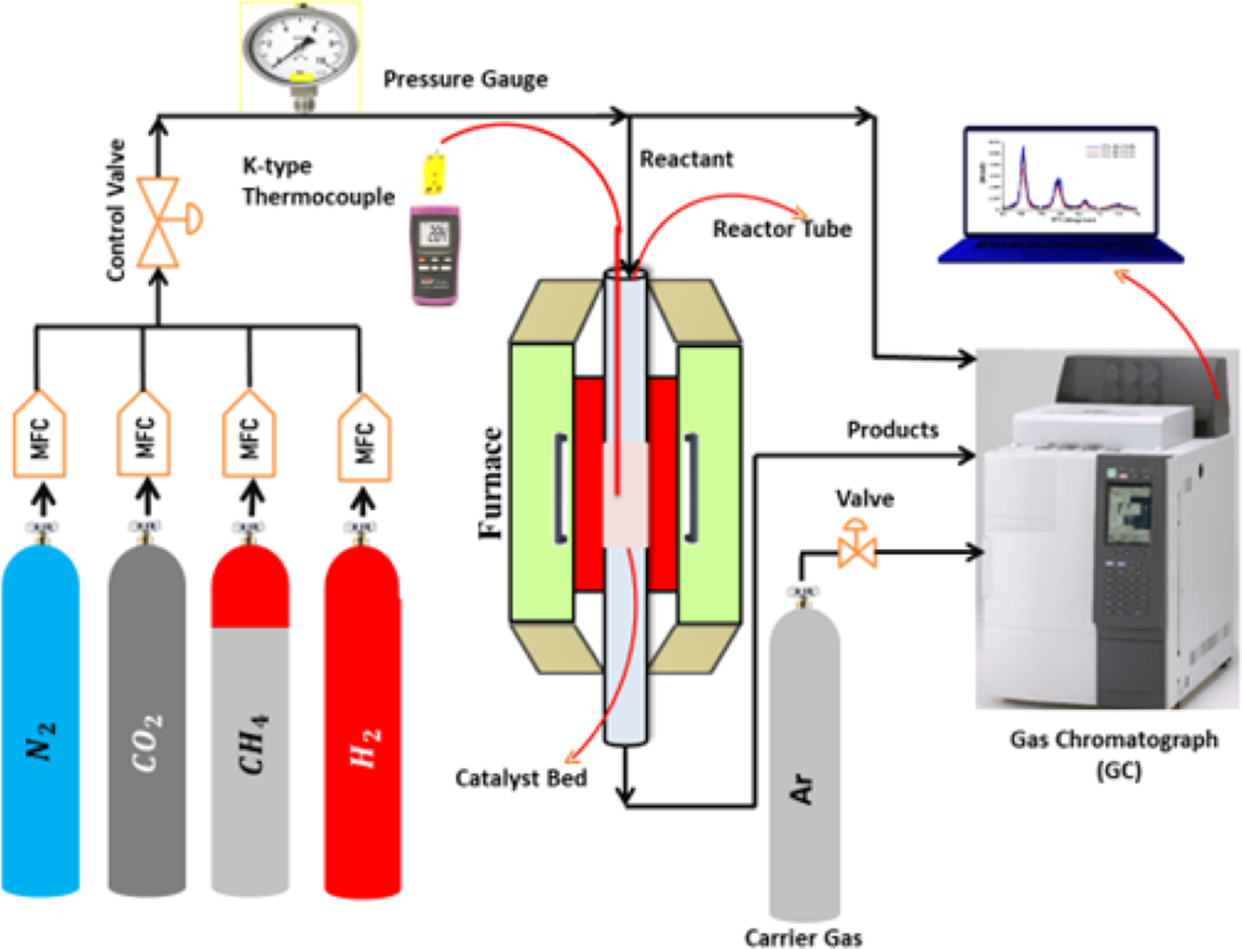
Experimental setup for conducting methane reforming.

At a pressure of 1 atm, the activity of the catalyst (0.1 g) was tested in a continuous-flow fixed-bed reactor. The reactor was 30 cm long and 0.94 cm in diameter. A thermocouple was attached to the middle of the catalyst bed to monitor the reaction temperature. Before the start of the reaction, hydrogen was introduced into the catalysts at a flow rate of 30 mL/min for an hour at 800 °C. Next, the reactor was purged with flowing nitrogen at a rate of 20 mL/min for 20 min at 700 °C to remove any remaining H_2_ inside the reactor. The reactor temperature was allowed to increase to the desired reaction temperature before the N_2_ purge was stopped. Throughout the process, a volume ratio of 30/30/10 was used to supply and maintain the feed gas (CH_4_/CO_2_/N_2_) at a rate of 70 mL/min at a space velocity of 42,000 mL/(hg_cat_). For theoretical simulation investigation, the temperatures of the process reaction are varied from 750 to 850 °C. The gas hourly space velocities are changed from 22,000 to 48,000 mL/(hg_cat_), while the ratios are adjusted from 0.5 to 1.0. A thermal conductivity detector and online GC were used to measure the concentrations of products and unconverted feed gases from the reactor. The experimental setup in this study is displayed in Figure 1.

## 3 Results

### 3.1 BET Evaluation

**Figure 2:**
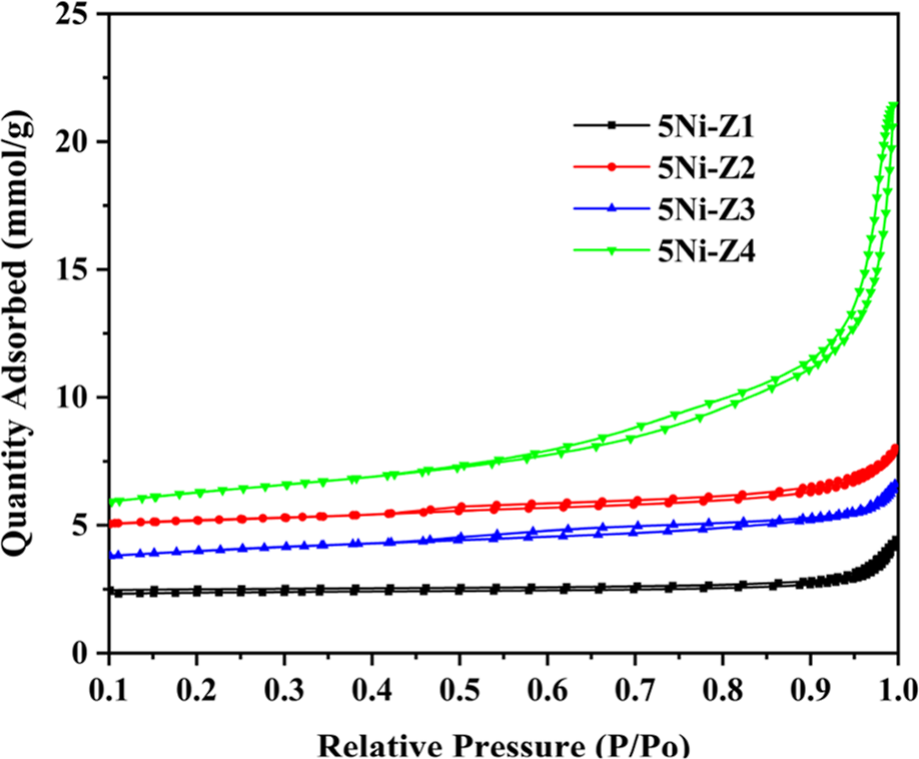
N_2_ measurements of adsorption and desorption on fresh Ni-supported catalysts.

N_2_ adsorption–desorption experiments were used to investigate the textural characteristics of the fresh catalysts, as illustrated in Table 1. According to the IUPAC classification, the isotherms in Figure 2 are type IV with H3 hysteresis loops, which proves that it is a mesoporous material according to the IPUAC classification.^11^ The typical H3-type hysteresis loop indicates the presence of slit-like pores in the zeolites. The N2 uptake started at a relative pressure range of 0.7–1.0. The sample with the highest surface area (5Ni/Z4) adsorbed the most N2 (455 cm^3^/g), while the sample with the lowest surface area (5Ni/Z1) adsorbed the least N2 (164 cm^3^/g).

**Table 1:** Texture-Related Characteristics of Newly Prepared Catalysts Supported by Nickel^a^

support/catalysts	surface area, BET (m^2^/g)	pore volume (cm^3^/g)	pore diameter (nm)	NiOa crystallite size (nm)
Z1	324	0.06	10.1	-
5Ni/Z1	164	0.07	16.3	15.4
Z2	361	0.12	7.8	-
5Ni/Z2	352	0.13	8.3	12.7
Z3	305	0.14	5.2	-
5Ni/Z3	286	0.12	6.1	20.0
Z4	471	0.61	13.5	
5Ni/Z4	455	0.58	13.8	20.1

a NiOa diameter = determined using Debye Scherrer equation.

The textural characteristics of the catalysts, such as the BET surface area, pore volume, and pore size, are listed in Table 1. Ni was added to the support, which had little effect on pore volume and led to a large reduction in surface area but a minor increase in pore diameter. The Ni particles partially obstruct the zeolite support’s pores for the decreased surface area. As a result, there is less surface area available overall for reactant molecules to interact with the catalyst. For the pore volume, the small alteration could suggest that Ni occupies the existing pores in the zeolite structure without significantly collapsing it; upon incorporation of 5 wt % Ni over Z1, the surface area is decreased by about half, whereas if the same amount of Ni is added over Z2, Z3, or Z4, the decrease in surface area is just 2.6–6% of the support area. It indicates lower dispersion of Ni up to SiO_2_/Al_2_O_3_ = 13 (in 5Ni-Z1), and fine dispersion of Ni is at SiO_2_/Al_2_O_3_ > 10 (in 5Ni-Z2, 5Ni-Z3, and 5Ni-Z4). A decrease may further hinder reactant accessibility in usable pore capacity, which could occur depending on the size and distribution of Ni particles as the increased pore diameter raises the possibility that certain smaller zeolite pores are being selectively blocked by the Ni particles.^12,13^ Out of all of the catalysts, 5Ni-Z4 has the largest surface area (554 m^2^/g) and pore volume (0.58 cm^3^/g), whereas 5Ni-Z1 has the smallest surface area (164 m^2^/g) and pore volume (0.07 cm^3^/g). A catalyst with a larger surface area and pore volume presents a wider region for the adsorption of reactant molecules.

### 3.2 Temperature Programming Reduction

**Figure 3:**
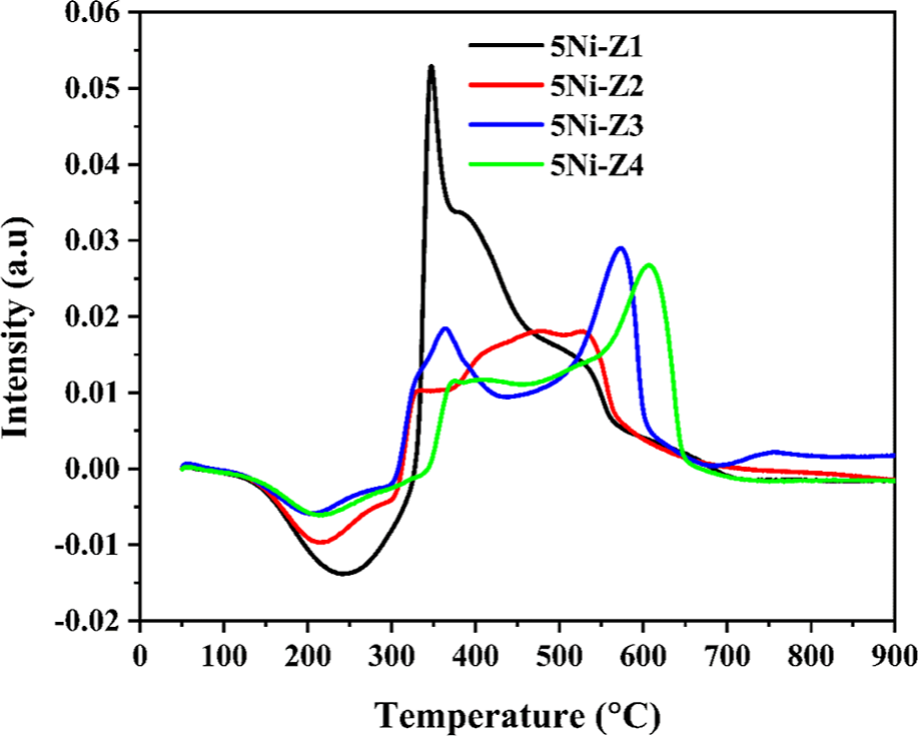
TPR measurements of the fresh Ni-supported catalysts calcined at 600 °C.

H_2_ temperature-programmed reduction was conducted to unravel the metal–support interaction of the catalysts, as displayed in Figure 3. The H_2_-TPR profile can be divided into two regions of reduction peaks of NiO species with different strengths of interaction with the support. The first region occurs at the low-temperature range of 300–400 °C and a high-temperature peak in the range of 450–550 °C. The metal–support interactions and peak temperatures may be linked as the lower peak temperature suggests that NiO and the support interact less strongly. This might be the result of a physical barrier separating the support material and NiO, or it could be the result of the support material’s weak affinity for NiO. Less strong contacts could make reduction easier but also decrease the stability of the active metal sites.

On the other hand, the greater peak temperature suggests that NiO and the support are interacting more strongly. This might be a result of the support material’s strong attraction for NiO or the chemical connections that are formed between them. More energy may be needed to activate the metal sites for catalysis, yet stronger contacts may result in a catalyst with greater thermal stability. The reduction of NiO beyond 650 °C is negligible. The negative peaks around 200 °C is possibly caused by hydrogen seeping into the mesopores.^4^ The quantity of hydrogen consumed during the TPR is shown in Table 2. The 5Ni-Z1 catalyst has a maximum amount of H_2_ consumption (20.498 cm^3^/g), indicating the presence of the highest number of reducible species of NiO over the catalyst surface. The H_2_-TPR profile envelops an intense peak at low temperature, and the other two merge peaks in the intermediate- and high-temperature range. It indicates the prominent presence of NiO species having weak metal support interaction among other NiO species having stronger interaction. The 5Ni-Z3 catalyst has a minimum amount of H_2_ consumption (12.791 cm^3^/g), indicating the presence of the least number of reducible species of NiO. It must affect the catalytic activity during the DRM. 5Ni-Z2 and 5Ni-Z4 have about equal amounts of H_2_ consumption (15–16 cm^3^/g), and both have NiO under weak as well as strong interactions with the support. It indicates that Ni derived from the reduction of NiO (under strong interaction with support) will be stable against high-temperature DRM reaction. In the mean of 5Ni-Z2, the high-temperature reduction peak is extended up to 600 °C, whereas, over 5Ni-Z4, it is extended to 650 °C. 5Ni-Z4 has the strongest metal–support interaction compared with the rest of the catalysts, but a very strong interaction of NiO with support (over 5Ni-Z4) may lead to difficulty in the reduction of NiO under the H_2_ stream, which may affect the catalytic activity results during the DRM reaction.

**Table 2:** Measurement of the TPR Analysis’s Hydrogen Consumption

zeolite-sample	temperature (°C)	quantity of H_2_-consumption (cm^3^/g)	total H_2_-consumption (cm^3^/g)
5Ni-Z1	347.23	20.498	20.498
5Ni-Z2	55.37	0.057	14.971
	477.15	9.669	
	525.74	5.245	
5Ni-Z3	56.09	0.050	12.791
	363.44	4.749	
	572.98	7.992	
5Ni-Z4	376.35	1.248	15.690
	405.30	3.0131	
	606.88	1.429	

### 3.3 XRD Analysis

**Figure 4:**
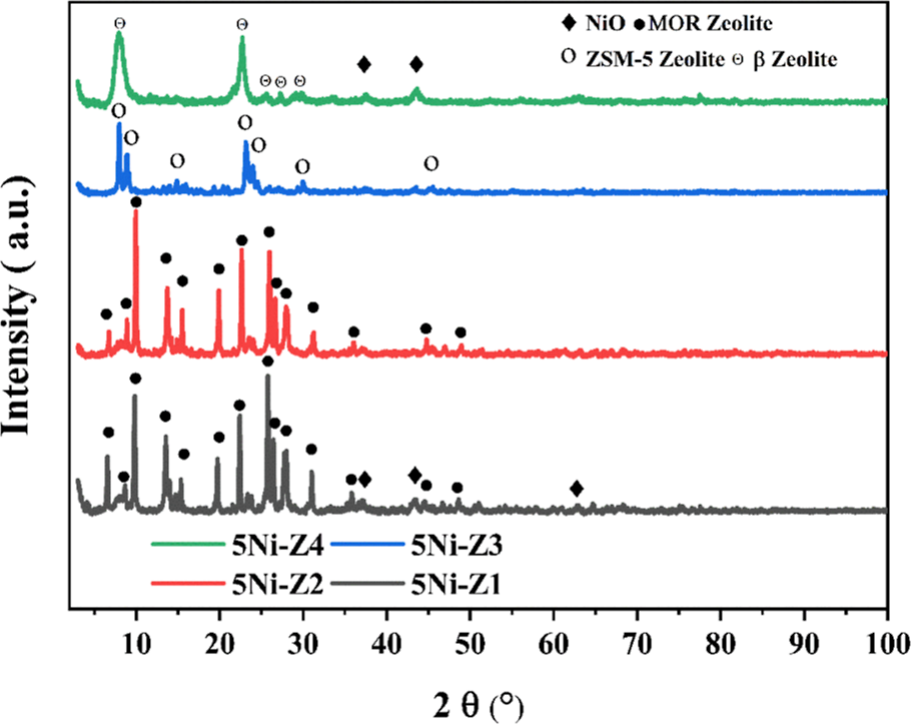
XRD patterns of fresh catalysts calcined at 600 °C.

XRD analysis, a technique that uses X-rays to investigate the crystalline phases within a material, is essential to understanding these catalysts. Based on the presence of their distinctive peaks, the researchers were able to validate the anticipated support materials for ZSM-5, mordenite, and zeolite beta by examining the patterns that were produced. The fresh crystals’ XRD patterns are shown in Figure 4. The supports used, Z1 and Z2, showed typical XRD diffraction patterns for mordenite, whereas Z3 and Z4 showed diffraction patterns resembling ZSM-5 and β-zeolites, respectively. For example, the ZSM-5 support material showed typical peaks at 2θ angles of 7.9, 8.8, 14.8, 23.2, 23.96, 29.9, and 45.3° [ICDS # 00-044-0002]. Similar to this, zeolite beta showed peaks at 2θ of 7.8, 22.6, 25.2, 27.1, and 29.6° [ICDS no. 00-048-0074], and mordenite showed peaks at 2θ of 6.5, 8.7, 9.7, 13.5, 15.3, 19.7, 22.4, 25.7, 26.4, 27.6, 31.0, 35.8, 44.5, and 48.6° [ICDS # 00-043-0171]. In contrast to the other catalysts, 5Ni-Z4 appears to have stronger NiO peaks, indicating a possible larger concentration of this active metal. This could have an impact on the catalyst’s catalytic activity. In summary, the XRD analysis verified the effective synthesis of the catalysts with the intended support materials and active NiO component and also hinted at a possible variation in the NiO concentration among the catalysts that could impact their efficacy. Table 1 presents the crystallite sizes of NiO among the catalysts. The size of the NiO crystallites varies between different samples (5Ni/Z1–5Ni/Z4), ranging from 12.7 to 20.1 nm. This variation could be due to the interaction of NiO with the support material. Crystallite size can influence the material’s properties, particularly its catalytic activity. Often, smaller NiO crystallites offer a larger active surface area for reactant molecules to interact with. This can potentially lead to a higher catalytic activity. The XRD data show no discernible peak shifts between the various catalysts. Nonetheless, as 5Ni-Z4 indicates, the observed intensity rise is connected to the rising silica–alumina ratio.

**Figure 5:**
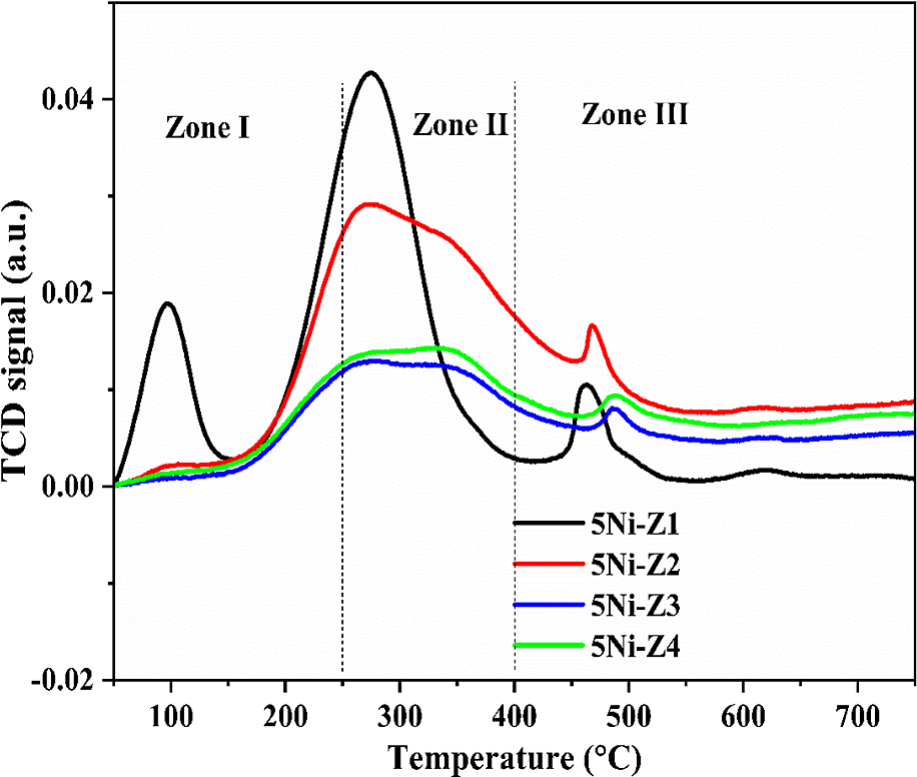
CO_2_-TPD measurements of the fresh Ni-supported catalysts calcined at 600 °C.

Figure 5 shows the CO_2_-TPD profile, whereas Table 3 displays the total CO_2_ consumption of all catalysts. The nature of catalyst support demonstrates a remarkable effect on the catalytic performance.^6^ Since the support with acidic nature increases the coke deposition, the basic sites increase CO_2_ adsorption. The CO_2_-TPD profile peaks appeared in three zones. A peak is found at a lower temperature region (∼250 °C) for the interaction of CO_2_ with the weak Brønsted basic sites. The peaks appearing in the temperature range of 250–400 °C are attributed to CO_2_ interacting with the oxygen anion (medium-strength basic sites), while peaks occurring above 400 °C are attributed to strong basic sites linked to adsorption on low-coordination oxygen anions acting as strong basic sites.^32^ Ni dispersed over mordenite-type zeolite framework (5Ni-Z1 and 5Ni-Z2) has higher CO_2_ desorption (or higher basicity) than Ni supported over ZSM and β-zeolite (5Ni-Z3, 5Ni-Z4). Among all catalysts, 5Ni-Z1, having the minimum SiO_2_/Al_2_O_3_ ratio (13), bears the highest concentration of surface hydroxyl Bronsted sites (weak basic sites), the highest concentration of surface oxygen anions (moderate strength basic sites), and a diffuse concentration of low coordination oxygen anions (strong basic sites). Upon increasing SiO_2_/Al_2_O_3_ to 20 (it is a 5Ni-Z2 catalyst), the basicity due to surface hydroxyl is lost. The other two catalysts, 5Ni-Z3 and 5Ni-Z4, have similar but inferior basicity profiles in the moderate strength to strong basic sites range.

**Table 3:** Measurement of CO_2_-Desorption Using the CO_2_-TPD Analysis

zeolite-sample	temperature (°C)	quantity of CO_2_-desorption (cm^3^/g)	total H_2_-consumption (cm^3^/g)
5Ni-Z1	097.81	1.28	7.19
	274.98	5.52	
	462.57	0.39	
5Ni-Z2	093.94	0.03	5.58
	268.89	4.74	
	468.09	0.14	
	869.98	0.67	
5Ni-Z3	273.04	2.75	3.35
	485.48	0.11	
	889.85	0.49	
5Ni-Z4	278.97	2.88	3.16
	486.11	0.14	
	892.13	0.14	

### 3.4 Catalyst Performance

**Figure 6:**
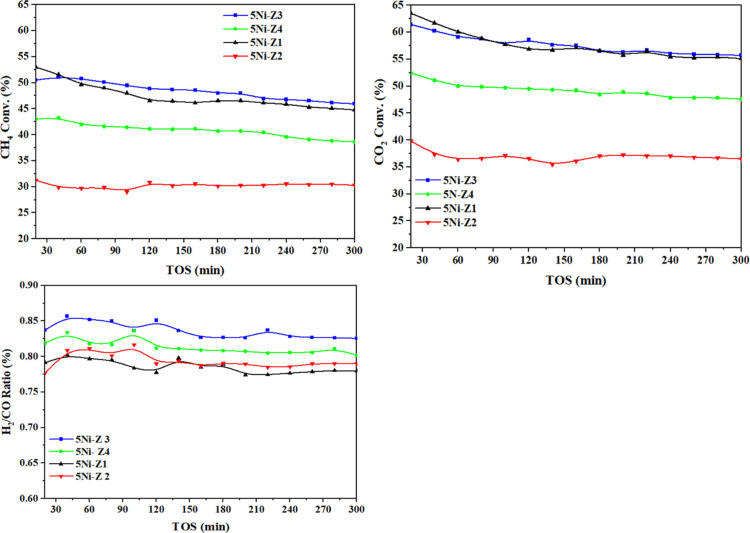
Conversions of CH_4_ andCO_2_, and H_2_/CO as a result of TOS [reaction circumstances: CH_4_/CO_2_/N_2_ = 3/3/1 (v/v/v); GHSV = 42,000 mL/gcat/h; *M*_cat_ = 0.1 g; *T* = 800 °C].

Before assessing the premade catalysts, a blank experiment was conducted using the same feed ratio and a specified reaction temperature (800 °C) but without the use of catalysts in an empty stainless-steel reactor. The blank’s CH_4_ and CO_2_ conversions at 800 °C were found to be 1.6% and 0.15%, respectively, while the H_2_/CO ratio was almost 0.12, indicating the thermal decomposition of CH_4_.^33^Figure 6 displays profiles of the H_2_/CO ratio as well as CH_4_ and CO_2_ conversions. At 800 °C, the activity of 5Ni/MS catalysts, where MS refers to the types of zeolites Z1, Z2, Z3, and Z4, was assessed. An induction phase of activation exists for all of the catalysts. The 5Ni-Z3 catalyst gave the highest DMR performance, with CH_4_ and CO_2_ conversions of 50% and 60%, respectively. The 5Ni-Z2 catalyst exhibited the lowest DMR performance, with CH_4_ and CO_2_ conversions of 30 and 38%, respectively, and an H_2_/CO ratio of 0.8. Results indicated that the zeolite support type affects the performance of the Ni-based catalysts in terms of both CH_4_ and CO_2_ conversion. The performance behavior of Z3 and Z1 is better than that of the other Z and Z4. For all catalysts, CO_2_ conversions are always higher than the corresponding CH_4_ conversions owing to the RWGS reaction (H_2_ + CO_2_ ↔ CO + H_2_O), in which the CO_2_ reacted with the produced hydrogen.^9^ Based on performance parameters, Table 4 presents the catalyst used in this work along with those found in the literature, indicating a promising degree of methane conversion.

**Table 4:** Performance Comparison for Methane Conversion

catalyst	temperature (°C)	WHSV (mL/(h·g_cat_)	CH_4_/CO_2_	CH_4_ conversion (%)	ref
Ni-SiCart	700	22,500	1:1	63	(17)
Ni/Mg-DML-180	700	60,000	1:1	63	(34)
2.5Ni-2Co-0.5Ru-1Ce-3Mg-3Zr–Al_2_O_3_	650	10,000	1:1	60	(20)
10Ni + 10Co/SBA-15	700	25,000	1:1	63	(21)
Ni/MgO-Al_2_O_3_	600	170,000	1:1	40	(23)
5Ni/MgO	700	42,000	1:1	40	(24)
Ni/MCM-41	700	36,000	1:1	50	(25)
5Ni/Halloysite Nanotubes	800	70,000	1:1	42	(26)
8Ni/Ce_*x*_Zr_1–*x*_O_2_–Al_2_O_3_–W	800	24,000	1:1	48	(27)
Ni/Pal-IM	800	27,000	1:1	58	(28)
5Ni-Z3	800	42,000	1:1	50	this work

### 3.5 Raman Evaluation

**Figure 7:**
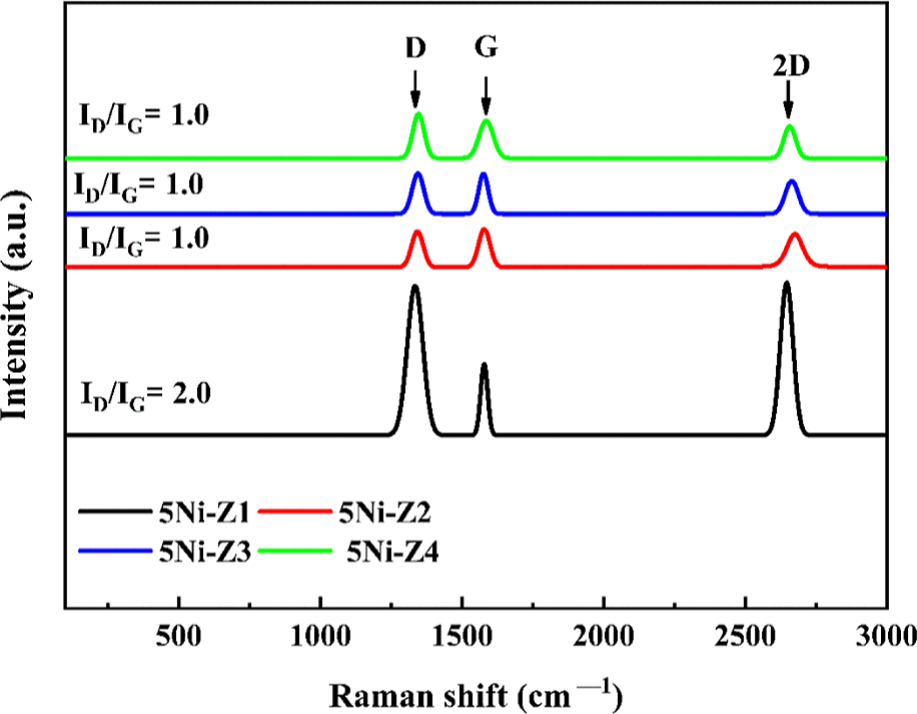
Raman spectra of the used Ni-supported catalysts at 800 °C.

Figure 7 displays the Raman spectra of the employed Ni-supported catalysts. All samples display three visible peaks. The position of the Raman shift bands that represent the molecular structure and composition is mostly independent of the type of support. The Raman shift bands at 1341–1354 cm^–1^ (D band) and 1597–1583 cm^–1^ (G band) can be identified. The G band is thought to be caused by in-plane carbon–carbon stretching vibrations in graphite, while the D band represents structural flaws in the material. Due to a combination of in-plane and out-of-plane vibrational modes, the 2D peak is a more complex characteristic. The number of graphene layers in a sample can be ascertained by comparing the relative intensities of the G and D peaks. The defect density in the graphene sample is determined by the *I*_D_/*I*_G_ ratio. A higher defect density is indicated by a higher *I*_D_/*I*_G_ ratio. *I*_D_/*I*_G_ ratio is found maximum (2) overspent 5Ni-Z1. Rest of the spent catalysts (5Ni-Z2, 5Ni-Z3, and 5Ni-Z4) have an equal *I*_D_/*I*_G_ ratio (1).

### 3.6 Temperature Gravimetric Analysis

**Figure 8:**
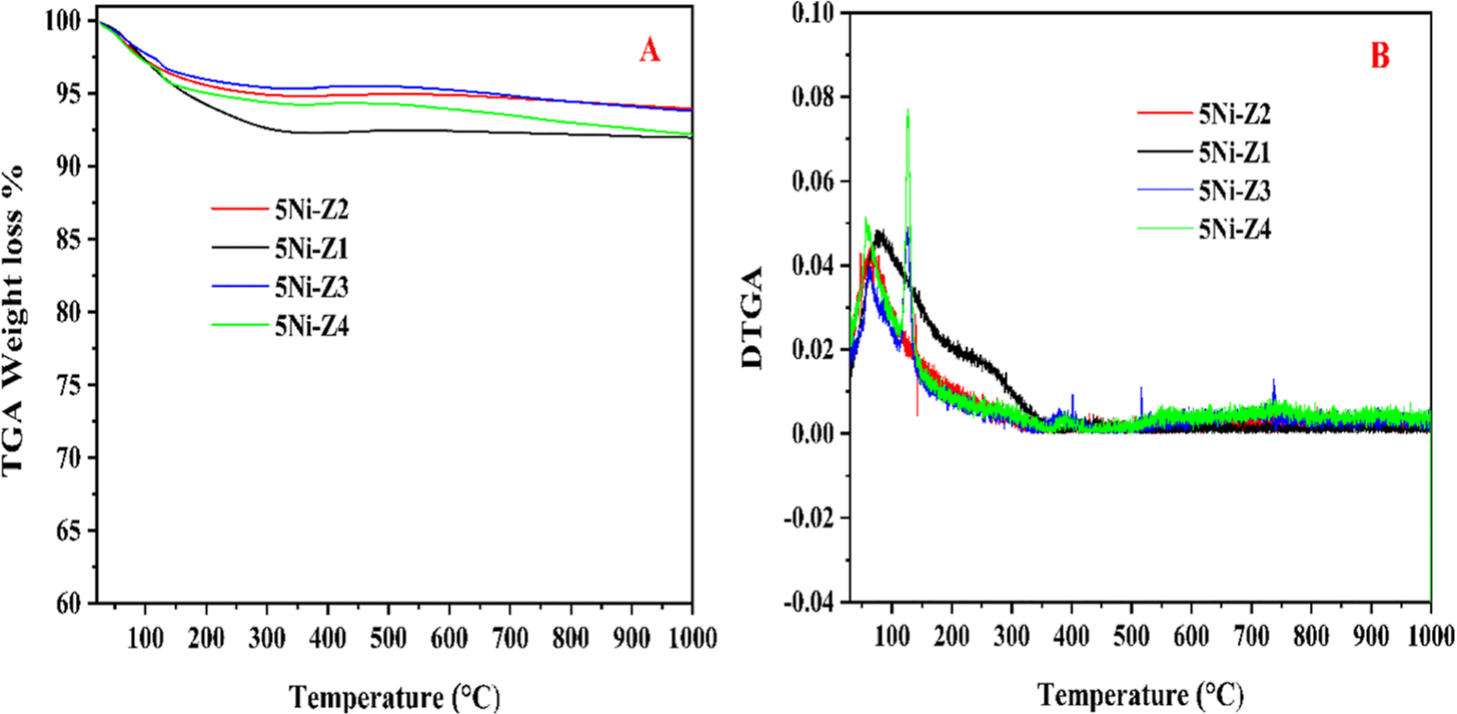
(A) TGA curve and (B) DTGA for TGA analysis of the used catalyst after 300 min time on stream.

Figure 8 displays the TGA and DTGA of the catalysts used, performed at 800 °C for 300 min on stream. Figure 8A exhibits the weight loss of the spent catalysts. TGA curve presents two segments: the weight loss between 0 and 250 °C is predominantly brought on by the removal of H_2_O and other chemisorbed species. The slight weight loss that occurred beyond 250 °C is due to the oxidation of all types of carbon. The recorded weight loss of all the catalysts ranged between 9 and 6%. Figure 8B displays the rate of change of TGA with temperature (DTGA). The catalyst samples show the highest weight loss occurs below 200 °C.

### 3.7 TEM Analysis

**Figure 9:**
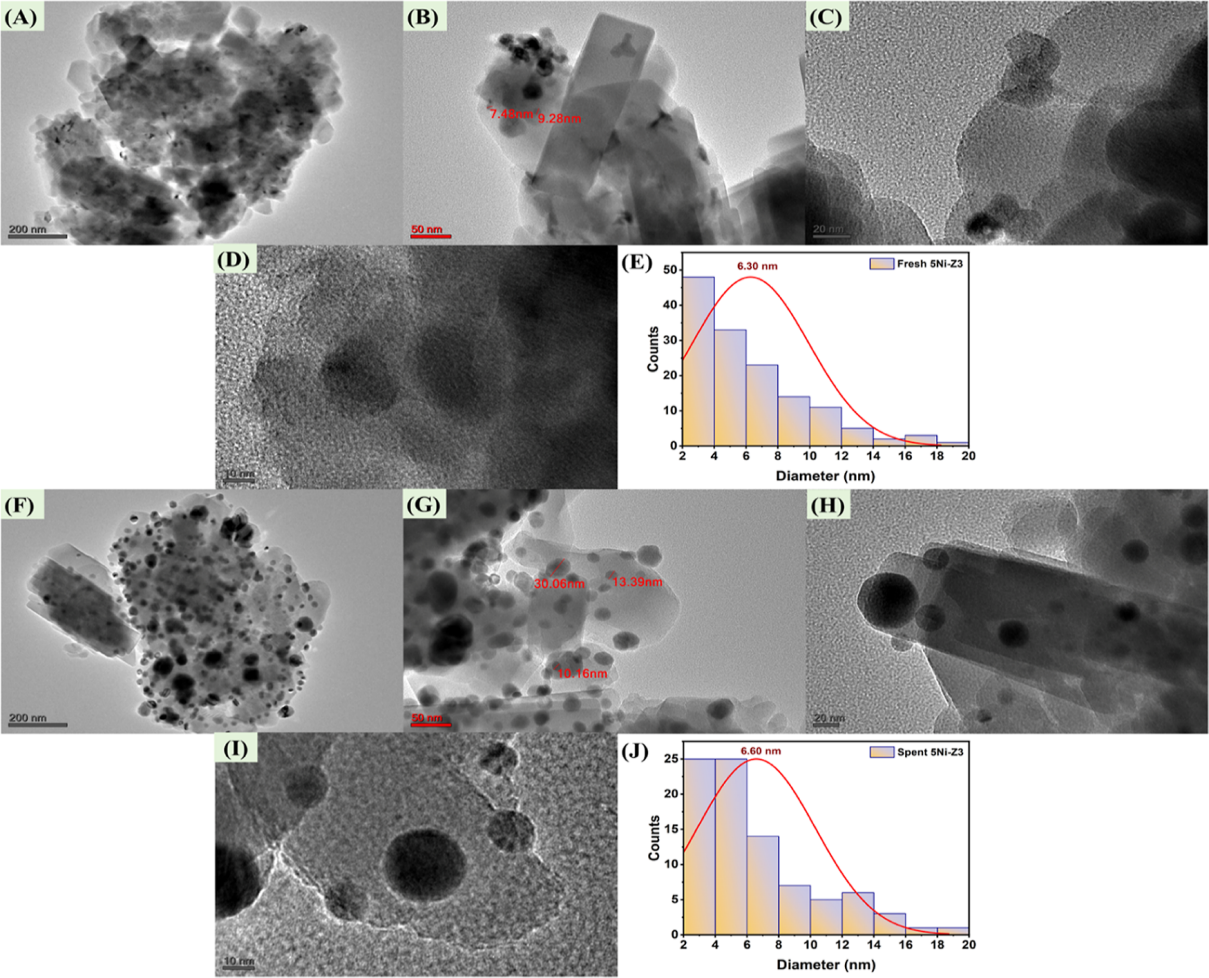
TEM images of fresh 5Ni-Z3 (A) at 200 nm scale, (B) at 50 nm scale, (C) at 20 nm scale, and (D) at 200 nm scale. (E) Particle size distribution of 5Ni-Z3. TEM images of spent 5Ni-Z3 (F) at 200 nm scale (G), at 50 nm scale (H), at 20 nm scale (I), and at 200 nm scale. (J) Particle size distribution of spent 5Ni-Z3.

The TEM images of fresh and spent 5Ni-Z3 catalysts at different scales are shown in Figure 9. The mean particle size of Ni is found to be 6.30 nm over the fresh catalyst and 6.60 nm over the spent catalyst. It indicates that the size of the Ni-particle is not affected much during the DRM reaction.

### 3.8 Mechanism of the Main Dry Reaction Pathway Adsorption

**Figure 10:**
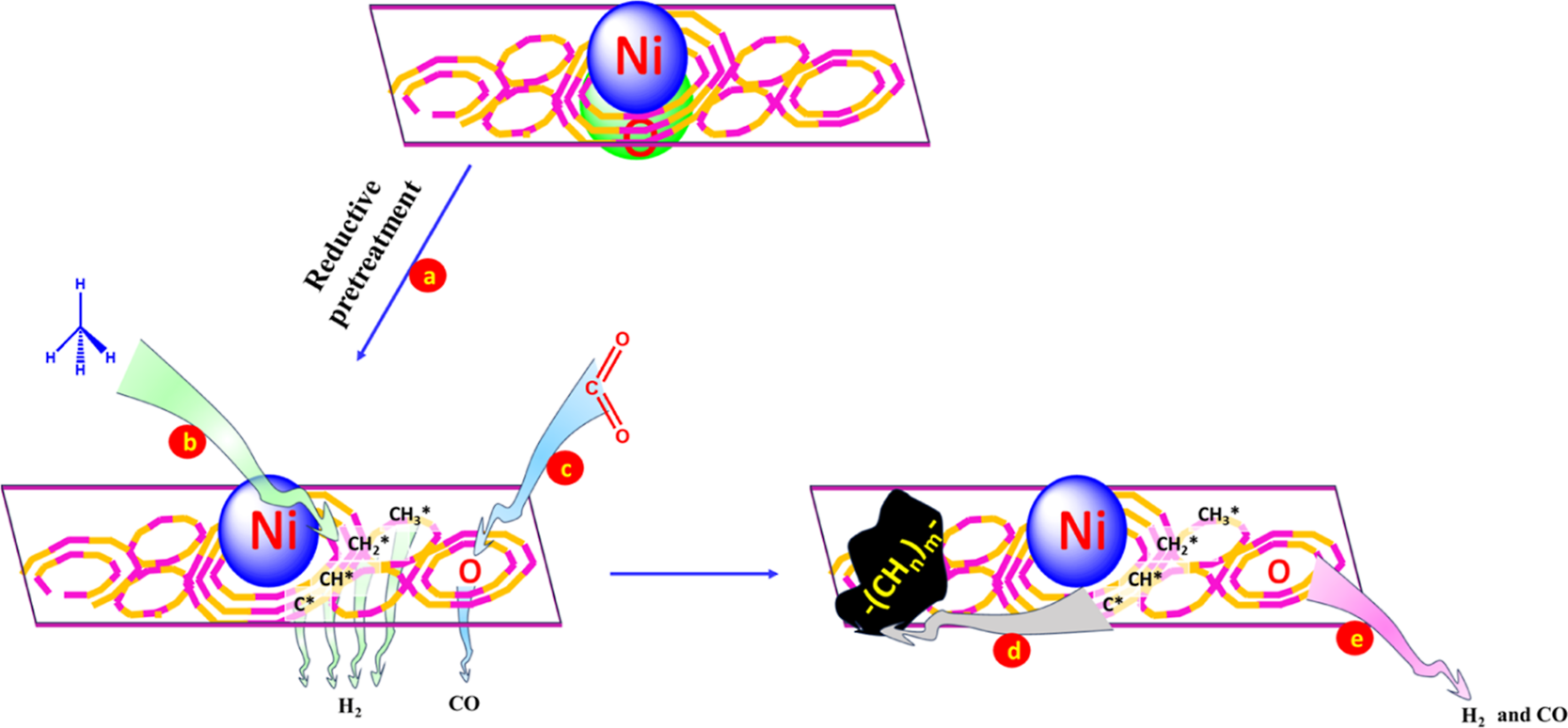
Mechanism diagram for the DRM process. (a) Reductive pretreatment. (b) Dissociation of CH_4_. (c) Dissociation of CO_2_. (d) Polymerization of CH_x_* (*x* = 3, 2, 1, and 0). (e) Reaction of CH_x_* (*x* = 3, 2, 1, and 0) and O*.

Figure 10 shows the mechanism diagram of the Ni-based catalyst supported over zeolite for the DRM process. Upon reductive pretreatment of the catalysts, the interacted-NiO species in zeolite is transformed into metallic Ni, which is a catalytically active site for DRM (Figure 10a). The C–H bond in CH_4_ breaks into CH_x_* (*x* = 3, 2, 1, and 0), typically at the Ni sites of the catalyst (Figure 10b). The CO_2_ molecule is also adsorbed at the basic sites and then dissociated into CO and O* (Figure 10c). The formed CO molecules were desorbed from the catalyst surface. The dissociated hydrogen generates H_2_ gas and, in the same way, the dissociated CH_x_* (*x* = 3, 2, 1, and 0) polymerizes and forms a carbon deposit (Figure 10d). The dissociated CH_x_* (*x* = 3, 2, 1, and 0) and O* interact and form CO and H_2_^15,16,22^ (Figure 10e).

### 3.9 Box–Behnken Designs

The Box–Behnken design (BBD) is a widely adopted experimental method, particularly effective in optimizing processes involving three factors. It is recognized for its efficiency in exploring response surfaces with fewer experimental runs compared with full factorial designs. BBD provides a structured approach to unraveling the intricate interactions among multiple variables. By systematically manipulating factors at predetermined levels within defined ranges, BBD empowers researchers to methodically evaluate the impacts of individual factors and their interactions with the response variable. Furthermore, the BBD, tailored for three-level factors, holds significant importance in the response surface methodology for fitting second-order models to responses. Originating from a fusion of two-level factorial designs with incomplete block designs, this design is crafted through a balanced incomplete block design comprising three treatments and three blocks.^35^ In the experimental design, the response variable is frequently represented through a polynomial equation of several factors. Utilizing the Taylor series expansion facilitates the derivation of this polynomial equation, approximating the response surface through a combination of linear, quadratic, and occasionally third-order terms.^36^ In our design, a quadratic polynomial is found, and the full quadratic model with three factors in the general form is given by eq 5
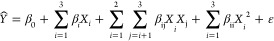
5where *X*_1_, *X*_2_, and *X*_3_ are the inputs in actual or coded values of the factors, β_0_ is the intercept coefficient, β_*i*_, *i* = 1, 2, and 3, are the linear coefficients, β_*ii*_ are the quadratic coefficients and β_*ij*_, *j* = 1, 2, and 3 are the interaction coefficients, and ε is the error term, which refers to the random variation in *Ŷ* that is not explained by the process parameters.^37^Table 5 lists the actual and coded values of the studied factors.

**Table 5:** Actual and Coded Values for the Process Parameters

	Levels	
process parameter	–1 (low)	+1 (high)
gas hour space velocity (ccg^–1^ h^–1^)	30,000	48,000
temperature (°C)	800	850
CH_4_/CO_2_	0.5	1

#### 3.9.1 Process Modeling and Analysis of Variance

**Figure 11:**
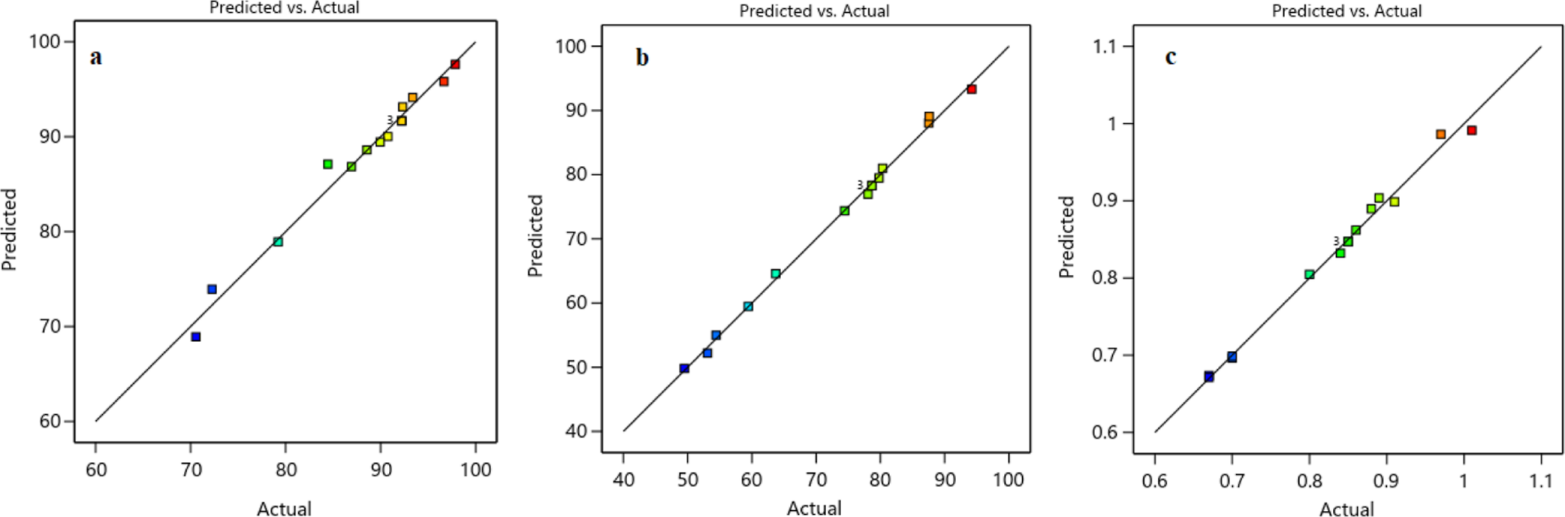
Experimental and model-predicted data for (a) CH_4_ conversion, (b) CO_2_ conversion, and (c) H_2_/CO ratio.

The statistical method ANOVA (Analysis of Variance) distributes the total variability in the data into components of different sources, as shown in Table 6. It helps to determine the significance factors and their interactions, thereby guiding the refinement of the model. The BBD method is applied to optimize processes, understand the factor interactions, and identify the best power transformation for the response data to normalize or equalize its variance.^38^ High *F*-values and small significance *P*-values indicate that the model terms are significant at approximately the 95% confidence level. High *R*^2^ values show that the model fits the experimental data well, as Figure 11 shows.^39,40^

**Table 6:** Analysis of Variance for the Various Components

source	sum of squares	d*f*	mean square	*F*-value	*p*-value
Response 1: CH_4_ conversion (*R*^2^ = 0.9824)					
model	910.88	7	130.13	55.85	<0.0001
A–A: temperature	375.38	1	375.38	161.12	<0.0001
B–B: ratio	221.34	1	221.34	95.01	<0.0001
C–C: CSV	133.82	1	133.82	57.44	0.0001
AB	54.83	1	54.83	23.54	0.0019
AC	42.19	1	42.19	18.11	0.0038
A^2^	46.63	1	46.63	20.01	0.0029
B^2^	42.64	1	42.64	18.30	0.0037
Response 2: CO_2_ conversion (*R*^2^ = 0.9972)					
model	2652.95	8	331.62	267.53	<0.0001
A–A: temperature	231.77	1	231.77	186.98	<0.0001
B–B: ratio	2142.83	1	2142.83	1728.71	<0.0001
C–C: CSV	34.32	1	34.32	27.69	0.0019
AB	31.30	1	31.3	25.25	0.0024
AC	31.87	1	31.87	25.71	0.0023
BC	9.80	1	9.80	7.90	0.0307
A^2^	43.75	1	43.75	35.30	0.001
B^2^	137.51	1	137.51	110.94	<0.0001
Response 3: H_2_/CO (*R*^2^ = 0.9923)					
model	0.1514	6	0.0252	172.89	<0.0001
A–A: temperature	0.0015	1	0.0015	10.36	0.0123
B–B: ratio	0.1352	1	0.1352	926.14	<0.0001
C–C: CSV	0.0066	1	0.0066	45.30	0.0001
AB	0.0030	1	0.0030	20.72	0.0019
BC	0.0012	1	0.0012	8.39	0.02
B^2^	0.0039	1	0.0039	26.42	0.0009

#### 3.9.2 Final Equations and Accuracy Models

According to the experimental data, using ANOVA at a level of significance (α = 0.05) and after identifying the significant factor effects and excluding the insignificant factor effects, and using Design-Expert software package version 13, the models have been suggested as follows
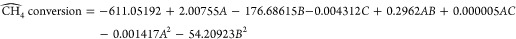
6
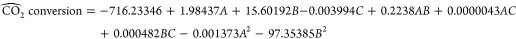
7
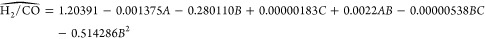
8where *A* is temperature, *B* is the feed ratio of reactants, and *C* is the CSV, the hourly space velocity. In the models, the constant or intercept coefficients represent the expected value of the response variable when all other factors are zero. The coefficients of the single factors represent the main effects of those factors, indicating the change in the response variable for a one-unit increase in each factor while holding all other factors constant. The coefficients of the products of two factors represent the effects of the interactions between them. Positive coefficients indicate a positive or increased effect on the response variable, while negative coefficients indicate the opposite. According to the coefficient of determination (*R*^2^), which evidence the goodness-of-fit of the model, the *R*^2^ values for the expected CH_4_ conversion, CO_2_ conversion, and H_2_/CO models are 0.9824, 0.9972, and 0.9923, respectively. This indicates that the models explain approximately 98.24%, 99.72%, and 99.23% of the total variations in the expected responses, respectively.

Table 7 presents the experimental and predicted values of the response variables based on the proposed model. As shown in Table 7, the closeness between the estimated responses from the proposed models and the actual values demonstrates the model’s ability to capture the observed data. This is evident from the small absolute percentage error rates of the three models, indicating that, on average, the predicted values have absolute percentage errors of 0.95%, 0.81%, and 0.78% compared to the actual values. A lower mean absolute percentage error (MAPE) value indicates a higher level of accuracy in the models. Equation 11 shows the MASE formula. Statistical tools, including *R*^2^ and various error metrics (APE, MAPE, and MAE), which are shown in eqs 9–11, are used to evaluate the model’s accuracy. Table 7 confirms a strong correlation between BBD models and experimental results with *R*^2^ near 1. This agreement is evident in Figure 11. Plotting predicted against experimental values is crucial for model assessment, with close alignment to the *X* = *Y* line indicating a good fit (see Table 8).
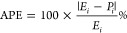
9
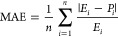
10
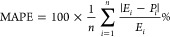
11

**Table 7:** Experimental and Predicted Data Results for Various Components of the Reaction System

Exp No	variables			Response								
				CH_4_ conversion			CO_2_ conversion			H_2/_CO		
	A/Temperature	B/ratio	C/CSV	EXP	Pred	%|error|	EXP	Pred	%|error|	EXP	Pred	%|error|
1	800	0.75	35,000	92.21	91.66	0.59	78.67	78.29	0.49	0.85	0.8471	0.34
2	800	0.75	35,000	92.21	91.66	0.59	78.67	78.29	0.49	0.85	0.8471	0.34
3	800	1	48,000	79.21	78.93	0.36	87.52	88.06	0.62	0.91	0.8988	1.23
4	750	1	35,000	70.56	68.92	2.32	78.08	76.96	1.43	0.89	0.9038	1.55
5	850	1	35,000	90.76	90.03	0.81	94.21	93.32	0.95	0.97	0.9863	1.68
6	800	0.75	35,000	92.21	91.66	0.59	78.67	78.29	0.49	0.85	0.8471	0.34
7	800	1	22,000	84.45	87.11	3.15	87.6	89.07	1.68	1.01	0.9913	1.85
8	750	0.75	48,000	72.27	73.93	2.30	63.71	64.58	1.36	0.8	0.8046	0.58
9	800	0.5	48,000	89.95	89.45	0.56	53.1	52.2	1.70	0.67	0.6738	0.57
10	750	0.75	22,000	88.55	88.61	0.07	74.43	74.37	0.09	0.86	0.8621	0.24
11	800	0.5	22,000	97.86	97.63	0.24	59.44	59.47	0.05	0.7	0.6963	0.53
12	750	0.5	35,000	86.93	86.85	0.10	49.5	49.82	0.64	0.7	0.6988	0.17
13	850	0.75	48,000	93.37	86.85	0.81	80.35	80.99	0.79	0.84	0.8321	0.94
14	850	0.75	22,000	96.66	95.81	0.88	79.78	79.49	0.37	0.88	0.8896	1.09
15	850	0.5	35,000	92.32	93.14	0.89	54.44	54.99	1.01	0.67	0.6713	0.19
					**MAPE**	**0.95**		**MAPE**	**0.81**		**MAPE**	**0.78**

**Table 8:** Comparison of Theoretical Model Predictions and Experimental Findings

goal’s function		variables						
		*T* °C	CSV mL/(hg_cat_)	CH_4_/CO_2_	CH_4_ conversion	CO_2_ conversion	H_2_/CO	desirability
max (CH_4_-conversion), max (CO_2_-conversion), and max (H_2_/CO)	criteria	value	value	value	max	max	max	
	optimum conditions theoretical	844.7	22000.2	0.939	92.73	89.52	1.00	0.899
	criteria	value	value	value	max	max	max	
	optimum conditions experimental	845.0	22000.0	0.940	92.05	90.10	1.00	

#### 3.9.3 Simulation of Design Expert Program

##### 3.9.3.1 One Factor Effect (2D) Plot

**Figure 12:**
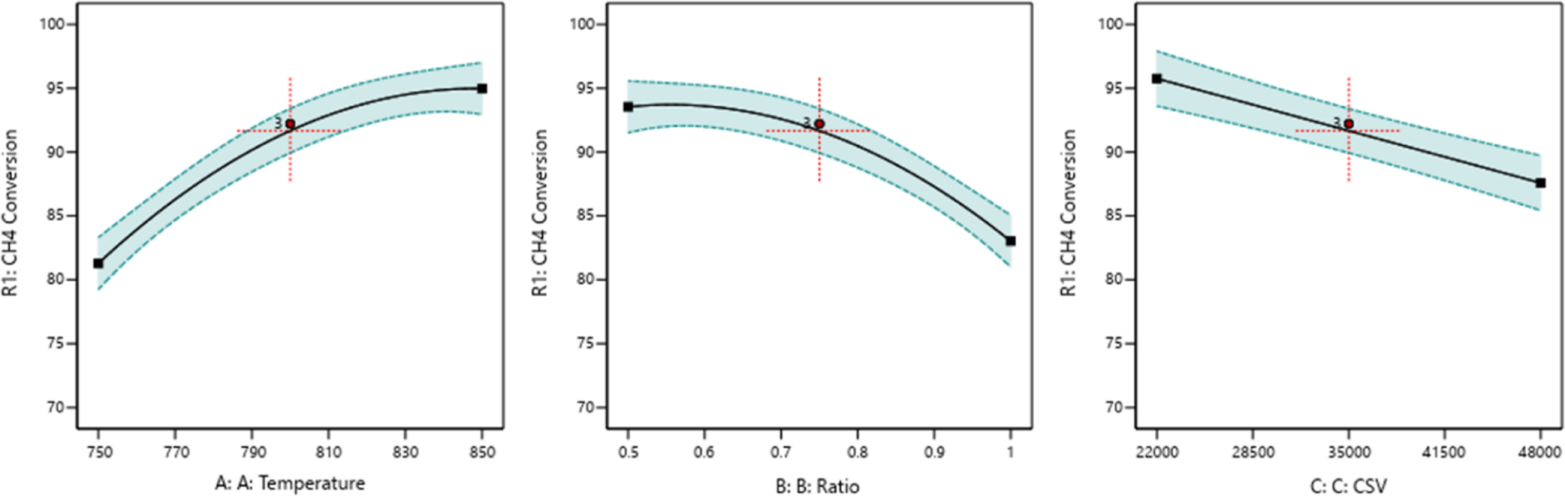
Relationship between the reaction parameters and CH_4_ conversion percentage.

**Figure 13:**
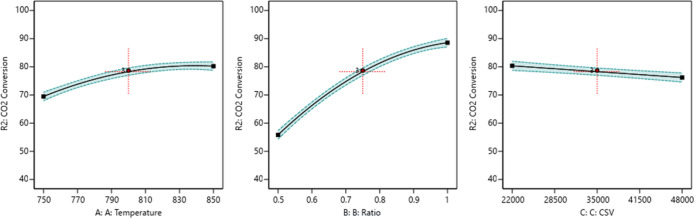
Relationship between the reaction parameters and CO_2_ conversion percentage.

**Figure 14:**
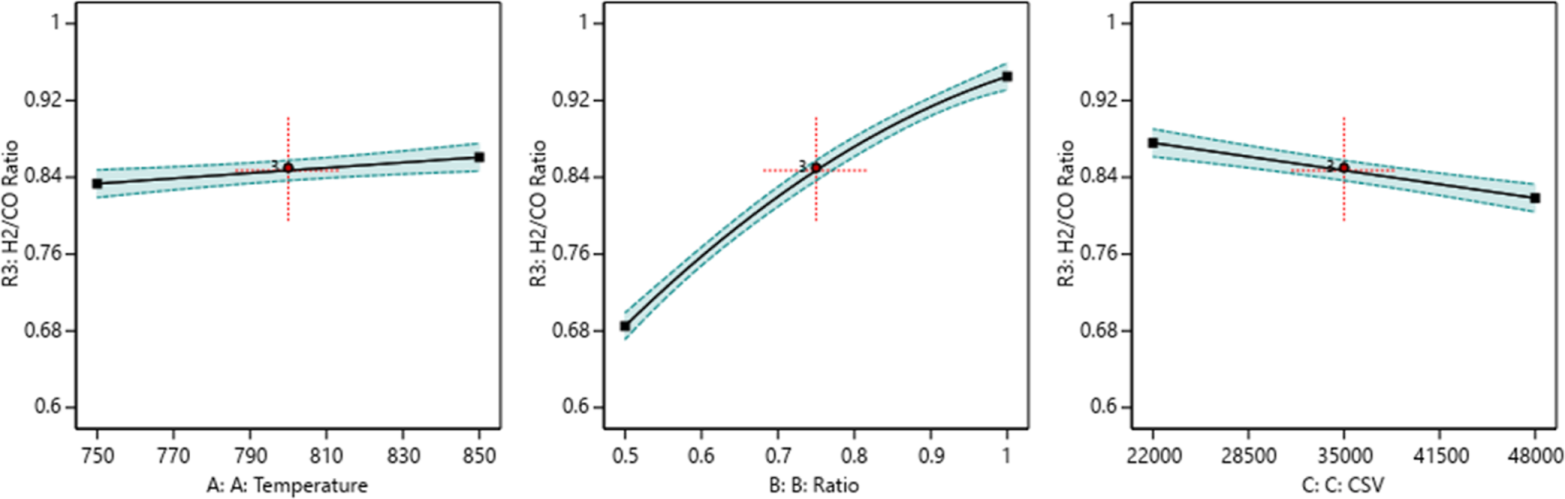
Relationship between the reaction parameters and the H_2_/CO ratio.

The effect of each process parameter on the reaction responses is shown in Figures 12–14.

Figure 12 indicates that increasing the temperature, decreasing the ratio, and decreasing the CSV value will increase the CH_4_ conversion. Figure 13 indicates that increasing the temperature, increasing the ratio, and decreasing the CSV value will increase CO_2_ conversion. Figure 14 indicates that increasing the temperature, increasing the ratio, and decreasing the CSV value will increase the H_2_/CO ratio.

##### 3.9.3.2 Two Factors Effect (3D Plot)

**Figure 15:**
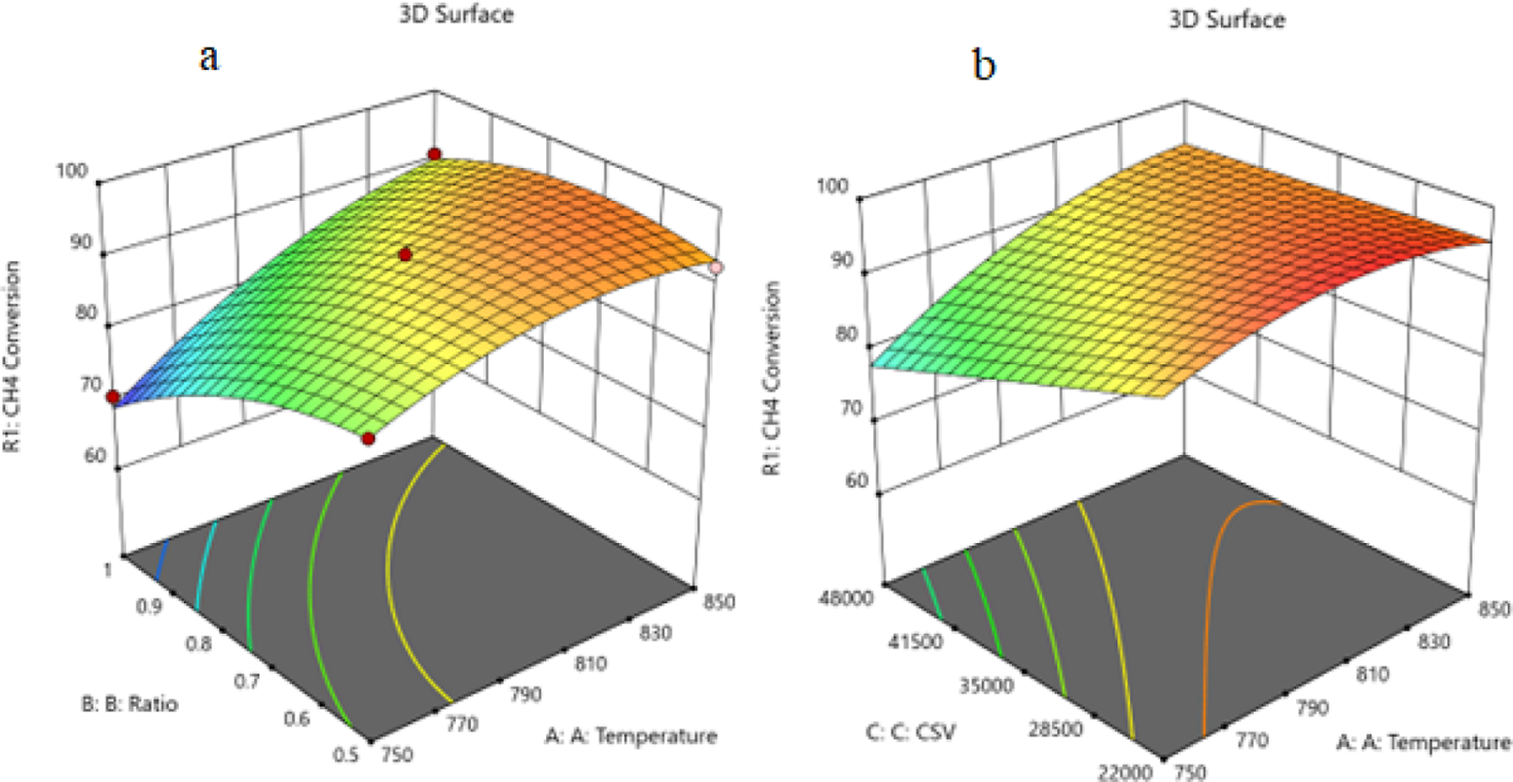
(a) Relationship between the temperature, ratio, and CH_4_ conversion % at CSV = 35000. (b) Relationship between the temperature, CSV, and CH_4_ conversion % at ratio = 0.605.

**Figure 16:**
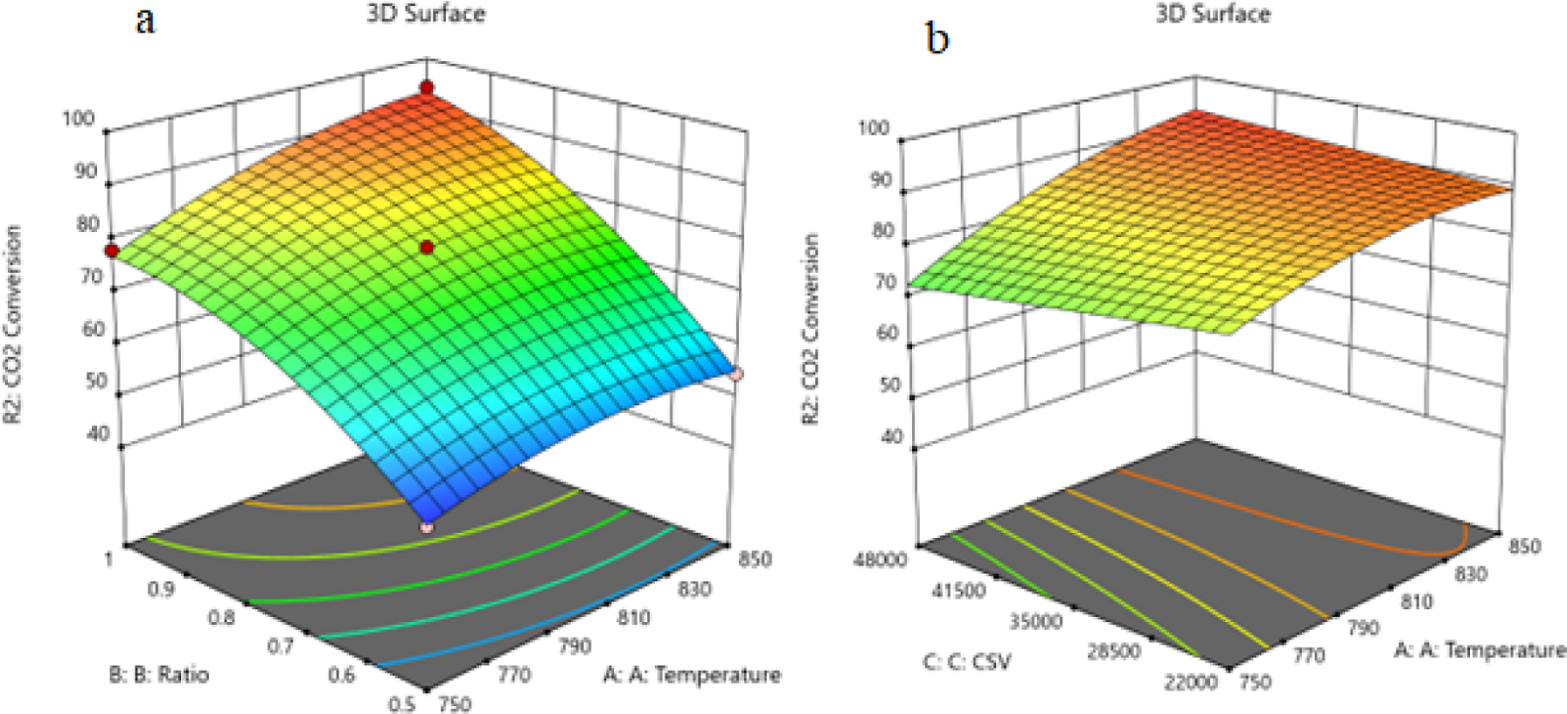
(a) Relationship between the temperature, ratio, and CO_2_ conversion % at CSV = 35,000. (b) Relationship between the temperature, CSV, and CO_2_ conversion % at ratio = 0.935.

**Figure 17:**
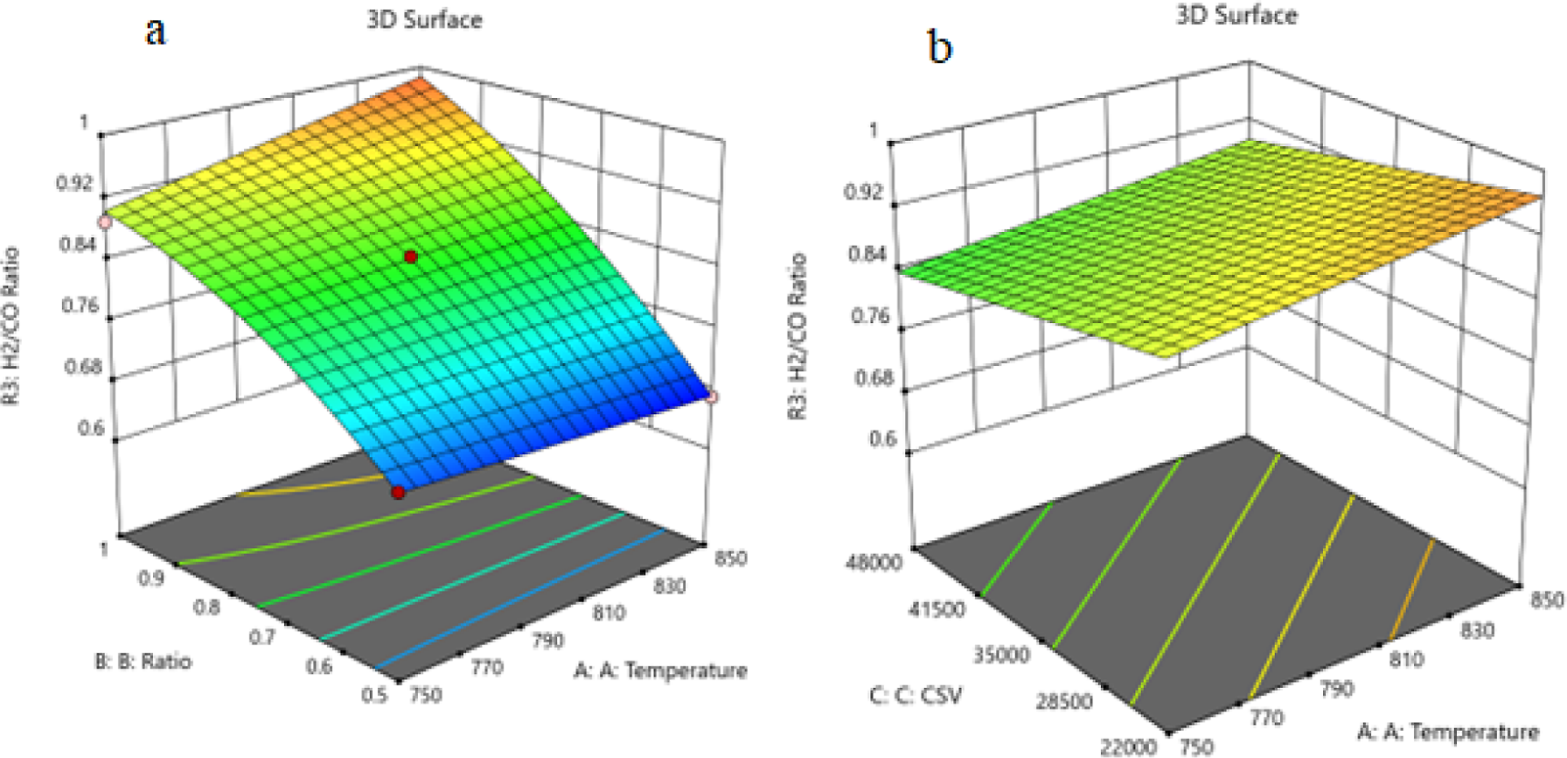
(a) Relationship between the temperature, ratio, and H_2_ /CO at CSV = 0.35000. (b) Relationship between the temperature, SV, and H_2_ /CO at ratio = 0.875.

Utilizing the regression parameters for different components yielded the following set of RSM equations representing the conversion or formation of various components within the reaction system expressed in terms of the actual independent variables. The equation in terms of actual factors enables predictions of responses at specified levels for each factor with the levels defined in their original units for accuracy. However, it is not suitable for assessing the relative impact of each factor due to coefficient scaling, and the intercept does not align with the center of the design space. Through the aid of the resulting equations and Design Expert program, the response surface plots were constructed for the predicted conversion or formation of the various components comprising the reaction system versus two process variables while keeping the third at a constant level or value, as shown in the 3D models in Figures 15–17.

Figure 15 shows the three-dimensional response surface plot, which represents the effects of the factors (temperature, CSV, and ratio CH_4_/CO_2_) on the variation of CH_4_ conversion. Figure 15a shows the surface plots, which represent the relationship between the response variable (CH_4_ conversion), and the two factors (temperature and the ratio CH_4_/CO_2_) at CSV = 35,000. It is shown that on increasing the temperature and decreasing the ratio of CH_4_/CO_2_, the CH_4_ conversion increases. Figure 15b shows the surface plots that represent the functional relationship between a designated response variable (CH_4_ conversion) and the two factors variables (temperature and CSV) with the CH_4_/ CO_2_ ratio fixed at 0.605. The response surface shows that with increasing temperature and decreasing CSV, the CH_4_ conversion increases. It was observed to increase from 70.56% at 750 °C to 97.86% at 850 °C. All factors have significant effects, but the temperature has a major effect on the variation of CH_4_ conversion. Figure 16 shows the three-dimensional response surface plot, which represents the effects of the factors (temperature, CSV, and ratio CH_4_/CO_2_) on the variation of CO_2_ conversion. Figure 16a shows the surface plots, which represent the relationship between the response variable (CO_2_ conversion) and the two factors (temperature and the ratio CH_4_/CO_2_) at CSV = 35,000. It is shown that by increasing the temperature and increasing the ratio CH_4_/CO_2_, the CO_2_ conversion increases. Figure 16b shows the surface plots that represent the functional relationship between a designated response variable (CO_2_ conversion) and the two factors’ variables (temperature and CSV) with CH_4_/CO_2_ fixed at 0.935. The response surface shows that with increasing the temperature and decreasing the CSV, the CO_2_ conversion increases. It was observed to increase from 49.5% at 750 °C to 94.21% at 850 °C. All factors have significant effects, but temperature has a major effect on the variation of the CO_2_ conversion. Figure 17 shows the three-dimensional response surface plot, which represents the effects of the factors (temperature, CSV, and ratio CH_4_/CO_2_) on the variation of H_2_/CO. Figure 17a shows the surface plots, which represent the relationship between the response variable (H_2_/CO) and the two factors (temperature and the ratio CH_4_/CO_2_) at CSV = 35,000. It is shown that on increasing the temperature and increasing the ratio CH_4_/CO_2_, the H_2_/CO ratio increases. Figure 17b shows the surface plots that represent the relationship between the response variable (H_2_/CO) and the two-factor variables (temperature and CSV) with CH_4_/CO_2_ fixed at 0.875. The response surface shows that with increasing temperature and decreasing CSV, the H_2_/CO ratio increases. It was observed to increase from 0.67% at 750 °C to 1.01% at 850 °C.

## 4 Discussion

This study investigates customized nickel–zeolite catalysts for addressing the problem of transforming methane, a powerful greenhouse gas, into syngas. Five catalysts with 5% nickel each were created and placed on different zeolite supports with silicon-to-aluminum ratios (Si/Al) ranging from 13 to 25. A variety of characterization methods was used, including temperature-programmed reduction, N_2_ adsorption–desorption, and XRD, to get a deeper understanding of the complex link between the catalyst’s structure and performance. The most important lesson to be learned from this study is how much the zeolite support affects the catalyst’s effectiveness. The Si/Al ratio and channel geometry are important factors in addition to the type of zeolite framework. The best catalyst, 5Ni-Z3, before SRM analysis had the highest conversion rates for carbon dioxide (60%) and methane (50%) at 800 °C, demonstrating this complex interaction. The homogeneous 10-ring channel structure and intermediate Si/Al ratio (15) of 5Ni-Z3 make it stand out. With the lowest concentration of active sites and a low basicity, this optimized architecture allows for enhanced reactant molecular dispersion throughout the catalyst, producing outstanding activity. On the other hand, 5Ni-Z2, which had mixed channel sizes (30% methane conversion and 38% CO_2_ conversion), performed the worst. It had large 12-ring and narrow 8-ring channels. This result is probably due to inert carbon deposits obstructing the channels, underscoring the need for channel regularity. It is interesting to note that 5Ni-Z4, which has the widest channels (12-ring) and similar basicity to 5Ni-Z3, produced findings that were between 5Ni-Z2 and 5Ni-Z3. The significant connection in 5Ni-Z4 between the nickel and the zeolite support explains this seemingly contradicting observation. The reduction of nickel oxide species, which is essential for the process to continue effectively, is hampered by the tight bond. The investigators did not end there. They used the response surface approach, a potent technique, in conjunction with numerical simulation to optimize syngas production utilizing the optimum 5Ni-Z3 catalyst to determine the ideal reaction temperature, space velocity, and feed ratio of methane to carbon dioxide. Through rigorous parameter optimization, the ideal syngas composition (over 92% methane conversion, above 89% CO_2_ conversion, and exact 1:1 hydrogen-to-carbon monoxide ratio) was reached at a reaction temperature of 845 °C. It is noteworthy that the 5Ni-Z3 catalyst produced results that nearly matched the theoretical predictions when the reaction was carried out under conditions that were similar to these predicted values. This remarkable agreement between experiment and theory provides compelling evidence of the efficacy of the determined operating conditions for the process of dry reforming. This extensive study concludes by emphasizing the vital role that specially designed zeolite supports and meticulously adjusted reaction conditions play in accomplishing the effective conversion of methane into useful syngas. Through the careful creation of catalysts and the application of potent optimization techniques, scientists are turning a potent greenhouse gas into a worthwhile clean fuel source, laying the groundwork for a more sustainable future.

## 5 Conclusions

This work investigated the effects of zeolite support structure on the performance of the Ni catalyst during 800 °C methane reforming. ZSM-5 (Z3, Si/Al 15) and beta (Z4, Si/Al 25) were contrasted with mordenite supports (Z1, Z2) that had different Si/Al ratios (13, 20). Due to its abundance of active sites, Z1 had the highest initial activity of 48% CH_4_ conversion; however, deactivation from carbon deposits in its small channels caused Z1 to malfunction. Because Z2 had fewer active sites and a greater Si/Al ratio, its 30% CH_4_ conversion activity was lower. Because of its ideal diffusion qualities, Z3, which has medium-sized pores, achieved a good balance between stability and activity of 50% CH_4_ conversion. Lastly, because of the strong metal–support interaction, Z4, which had the broadest channels, exhibited moderate activity. The most important factor, according to the response surface methodology study, was the reaction temperature, which was followed by space velocity. The projected yields of 92.73% CH_4_ conversion and 89.52% CO_2_ conversion at optimal conditions of 844.7 °C and 22,000.2 cm^3^ g–^1^ h–^1^ and a feed ratio of 0.939 substantially matched the experimental data. In summary, the performance of the Ni catalyst is greatly influenced by the zeolite support structure. Although Z1 had the highest initial activity, Z3 is a more plausible candidate due to its balanced activity and stability after Z1 was deactivated. Further optimization using RSM found conditions for optimizing CO_2_ and methane conversions while reaching a target hydrogen-to-carbon monoxide ratio.

## Data Availability

Data is contained within the article.
